# Effect of antioxidants on lipid oxidation in herring (*Clupea harengus*) co-product silage during its production, heat-treatment and storage

**DOI:** 10.1038/s41598-022-07409-8

**Published:** 2022-03-01

**Authors:** Mursalin Sajib, Markus Langeland, Ingrid Undeland

**Affiliations:** 1 Food and Nutrition Science, Department of Biology and Biological Engineering, Chalmers University of Technology, 41296 Gothenburg, Sweden; 2 Department of Animal Nutrition and Management, Swedish University of Agricultural Sciences, 75007 Uppsala, Sweden

**Keywords:** Biochemistry, Lipid peroxides

## Abstract

Provided high product quality, ensilaging can be used to valorize fish filleting co-products into a silage suitable for food applications. However, a documented challenge for products from hemoglobin-rich fish raw materials is the high susceptibility to lipid oxidation, calling for stabilization by antioxidants. In a comparison among different rosemary-containing antioxidants and isoascorbic acid, we here found that the commercial mixture Duralox MANC-213 (MANC) provided the best protection against peroxide value and 2-thiobarbituric acid reactive substances (TBARS) development during ensilaging of herring filleting co-products (0–7 days, 22 °C), and also during subsequent heat-treatment (30 min, 85 °C). Increasing MANC concentration from 0.25 and 0.75 to 1.25% lowered TBARS values from 43.53 and 25.12 to 18.04 µmole TBARS/Kg silage, respectively, after 7 days of ensilaging. During storage at 4 °C/22 °C in presence of MANC, 1.25% provided the highest protection with 87–90% and 66–73% lower TBARS, at 4 °C and 22 °C, respectively, at 6 months compared to the controls. At this time point, heat-treated silages had lower protein degree of hydrolysis and free amino acids values than the non-heat-treated one. Regardless of antioxidant addition, total volatile basic nitrogen (TVB-N) formation still increased during the storage, but, overall, TVB-N values in silages were below the acceptable limit of 30 mg TVB-N/100 g fish for human consumption. Together with lipid oxidation data, this suggest that herring silage produced in presence of antioxidants can be used both for high quality feed and food applications.

## Introduction

**Figure 1:**
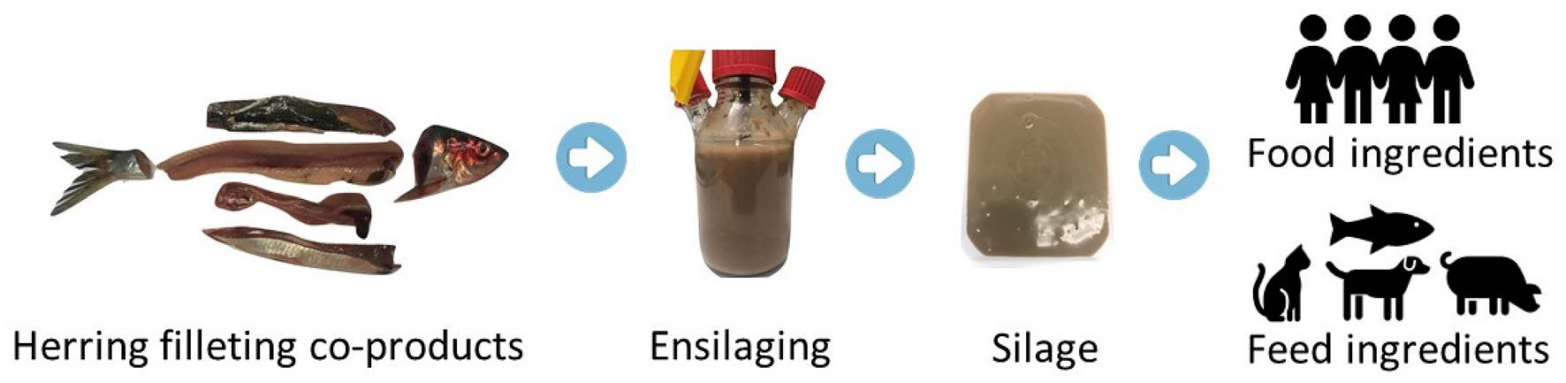
Ensilaging of herring filleting co-products; figure modified after Sajib et al.^5^.

The global demand for animal-derived protein is expected to double by 2050^1^; to meet this increasing demand we need to increase protein production, as well as maximize the use of our existing resources. However, our existing food production system is still inefficient and unsustainable in terms of e.g. resource use and environmental impacts, which costs around two trillion dollars globally considering the costs of e.g. losses in productivity, energy, natural resources, and, social and environmental costs^2,3^. For example, since around 70% of all caught/harvested fish is processed into convenience products e.g. fish fillets, around 20–80% co-products are generated^4^, which primarily goes to lower value applications like feed, for fur animals as mink, or fishmeal/fish oil production. In the worst case, co-products are discarded as waste. Given the ban on mink farming in 2020 to stop Covid-19 transmission, and the highly energy-demanding processes required to produce meal and oil, ensilaging—preservation under acidic conditions—could provide a cost-efficient process to valorize fish filleting co-products into high value peptide ingredients ensuring a circular approach (Fig. 1)^5^. This process can be considered as a “green” process because it does not generate any waste and does not comprises any harmful solvents^6^. Ensilaging protects the co-products against microbial spoilage and at the same time facilitates endogenous protease-mediated autolysis, resulting in a protein hydrolysate, usually known as silage^7^. Ensilaging has several advantages over fishmeal production, e.g. it can be done with small quantity of co-products where fishmeal plant is not economically viable, ensilaging tanks can be placed anywhere e.g. on fishing vessels where co-products are generated and the process is milder in the sense that it uses less heat, thereby avoiding negative side reactions as protein cross-linking^8,9^. Since fish processing co-products can be classified as category 3 co-products which are fit for human consumption^10^; fish co-product silage can be used not only for feed, but also for food applications. Silage can be added to food products in a similar way as fish protein hydrolysates are used today, e.g. as fortification of drinks, soups, sauces, dietary supplements, sports nutrition products etc. in a liquid, semi-dried or dried state^11^. However, one of the main challenges limiting higher value applications of silage is lipid oxidation taking place during ensilaging, rendering the silage rancid^12^.

Filleting co-products from pelagic fish like herring (*Clupea harengus*) contain around 4.6–17.9% crude lipids^5^, which are highly susceptible to oxidation due to its polyunsaturated fatty acid (PUFA) content and the abundance of blood-derived hemoglobin (Hb) in the herring tissue^13,14^. The acidic pH used in ensilaging (i.e. pH 3.50) changes Hb from its native oxyhemoglobin (oxyHb) to the methemoglobin (metHb) state, facilitating heme group release, which can then promote lipid oxidation via heme-mediated peroxide cleavage^15–17^. The negative consequences of lipid oxidation are well-known, e.g. a reduction in the sensorial quality^18^, reduced nutritional value^19^, and, in case of feed applications, a lower feed conversion ratio and reduced carcass quality have been observed in fish and broilers^8^. Also, an increased risk of tumor and atherosclerosis development has been observed in animal studies following ingestion of oxidized lipids^20^. Thus, to produce a high-quality silage—here referred to as “silage 2.0”—for value-added food/feed applications, the lipid oxidation reaction should be kept to a minimum, and preventative strategies like antioxidants should be added at the beginning of ensilaging.

Isoascorbic acid is a stereoisomer of ascorbic acid and has shown good effect as an antioxidant against Hb-mediated oxidation of minced herring co-products during ice storage (pH ~ 6.37)^21^. Its main mechanism of action is to scavenge free radicals and reduce hypervalent iron^22^. Rosemary (*Rosmarinus officinalis* L.) extract has a positive consumer image due to being a “natural” antioxidant^23^. It contains many phenolic compounds e.g. carnosol (picrosalvin), and carnosic acid (rosmaricine), and provides antioxidative protection by scavenging free radicals^24^. Although never before reported for use in silage, it has great potential since it has been reported to inhibit lipid oxidation both under acidic environment (pH 4.0–5.0)^25^ and heat-treatment (90 °C for 50 min)^26^. The latter is of importance in silage production since it will allow a better control over the extent of hydrolysis during subsequent long-term storage, and thus will result in a silage rich in short-chain peptides with potential bioactivity rather than free amino acids (FAA)^5,27^. Stopping the autolysis will also partly prevent total volatile basic nitrogen (TVB-N) formation, a spoilage indicator important for fish-derived products^28^. Further, heat-treatment will also mitigate pathogen risks^29^, as well as inactivate thiaminases^8^. To date, however, very little is known about the effect of heat-treatment on lipid oxidation at low pH (pH ~ 3.50), and the same is true for the effectiveness of antioxidants under such conditions.

The main aim of this study was to investigate the effect of selected rosemary-based antioxidants and isoascorbic acid at three different concentrations on lipid oxidation inhibition during production of silage from herring filleting co-products, as well as during its heat-treatment and subsequent storage at 4 °C and 22 °C for up to 6 months. Two well-known synthetic antioxidants, butylated hydroxytoluene (BHT) and propyl gallate, were also investigated. In addition, the effects of antioxidant additions, heat-treatment, and storage conditions on protein degree of hydrolysis (DH), FAA, and TVB-N formation in the silage were also investigated.

## Materials and methods

### Materials

Herring filleting co-products were kindly provided by Sweden Pelagic Ellös AB (Ellös, Sweden). The co-products—consisting a mix of heads, frames, tails, skins, guts and other intestinal organs—were from herring filleted on the 21st of October 2019 (batch 1) and 4th of September 2018 (batch 2), and were collected immediately after filleting and transported to the lab within the same day under cold storage (5 °C). Upon arrival, they were minced using a meat grinder (la Minerva, Italy) with a 4.5 mm hole plate, and stored in 500 g aliquots at − 80 °C until further use. Antioxidants used in this study were BORDANTIX LIQUID W/S and BORDANTIX LIQUID O/S (EVESA, Cádiz, Spain), Duralox MANC-213 (Kalsec, Kalamazoo, Mich., UK), isoascorbic acid (Sigma-Aldrich, USA), BHT (Sigma-Aldrich, USA), and propyl gallate (Sigma-Aldrich, USA). The latter two antioxidants were used in studies where co-products from batch 2 were used; otherwise, co-products from batch 1 were used in studies with the remaining antioxidants.

### Ensilaging of herring filleting co-products, heat-treatment, and storage of silages

Antioxidants were added to minced herring filleting co-products at 0.25, 0.75, and 1.25% w/w concentrations, and mixed for 10 min at 10 rpm using an overhead stirrer attached to the ensilaging reactors. Control refers to silage without any antioxidants. The co-products, with or without antioxidants, were then ensilaged in 500-mL glass reactors by adding 2.5% v/w formic acid (85% purity) with continuous stirring (10 rpm) at ambient temperature (i.e. ~ 22 °C) (Fig. 1) as previously described^5^. Samples were collected at 0, 1, 4, and 7 days, and, immediately put in 5-mL Eppendorf tubes for storage at − 80 °C until further use.

After 7 days, portions of silages were subjected to heat-treatment by moving around 10 mL silage into 15-mL tubes, and heating the silages at 85 °C for 30 min in a pre-heated water bath. Silages were then cooled to ambient temperature, homogenized, transferred to 5-mL Eppendorf tubes and stored at − 80 °C for further analysis, and to 15-mL tubes for storage trials as described below.

For the storage trials, around 10 mL of both heat-treated and non-heat-treated silages were stored in 15-mL tubes at 4 °C and 22 °C. Silage containing tubes were vortexed for 30 s before sampling at 0, 1, 3, and 6 months, and sub-samples were put in 5-mL Eppendorf tubes, which were immediately frozen and stored at − 80 °C until further use. Storage trials were performed with each silage type in triplicate tubes (n = 3).

### Determination of peroxide value (PV) and 2-thiobarbituric acid reactive substances (TBARS)

For the analyses of PV and TBARS, around 2 g of silage was mixed using 20 mL of ice-cold chloroform:methanol (2:1) containing 0.05% w/v BHT, followed by 8 mL of ice-cold 0.5% w/v NaCl addition, according to Lee et al.^30^. The mixture was then centrifuged at 3000×*g* for 6 min (4 °C), and the resulting lower and upper phases were then used for PV and TBARS analysis, respectively, according to Undeland, et al.^31^ (PV) and Schmedes and Hølmer^32^ (TBARS) using a spectrophotometer (Cary 60 UV–vis, Agilent technologies, USA).

### Determination of protein degree of hydrolysis (DH)

DH was measured according to Nielsen et al.^33^ with slight modifications as described in Sajib et al.^5^. Briefly, to 3.75 mL of o-phthaldialdehyde (OPA) reagent, 0.5 mL of pre-diluted silage sample was added, followed by 2 min incubation at ambient temperature, and the absorbance was then measured at 340 nm using a spectrophotometer (Cary 60 UV–vis, Agilent technologies, 117 USA).

### Determination of free amino acids (FAA)

Sample for FAA analysis was prepared as described by Sajib et al.^5^, and then analyzed by LC/APCI-MS according to a method described by Harrysson et al.^34^. Briefly, 0.9 g of silage was centrifuged at 12,000×*g* for 10 min (4 °C), where after 300 µL supernatant was mixed with an equal volume of 7.5% trichloroacetic acid (TCA) solution, kept on ice for around 15 min, followed by centrifugation as described earlier, and the supernatant was then used for analysis.

### Determination of total volatile basic nitrogen (TVB-N)

TVB-N was measured according to a method described by Sajib et al.^5^. Briefly, 2 g silage was extracted using 8 mL of 4% TCA solution, followed by centrifugation at 3000×*g* for 15 min, and then 2 mL supernatant was used for analysis using Conway cells.

### Statistical analysis

Results were expressed as mean values (n = 2 or 3) ± standard error of the mean (SEM). ANOVA analysis with Tukey Honest Significant Differences (HSD) was performed on R software (https://www.r-project.org/), and significant differences were accepted at p < 0.05. Selected data sets were also subjected to multivariate analysis using MODDE Pro software (version 12.1, Sartorius Stedim Data Analytics AB, Sweden). The purpose was to get an overview of how the studied factors and their interactions affected the responses. Full factorial (mixed) designs were used, and the models were fitted with partial least squares regression (PLS). All the responses were scaled and centered, and, the size of coefficients—i.e. half of the effect—represents the change in their respective responses when a factor varies from medium to high level, while keeping other factors at their average values.

## Results and discussion

### Effect of antioxidants on PV and TBARS inhibition during ensilaging

**Figure 2:**
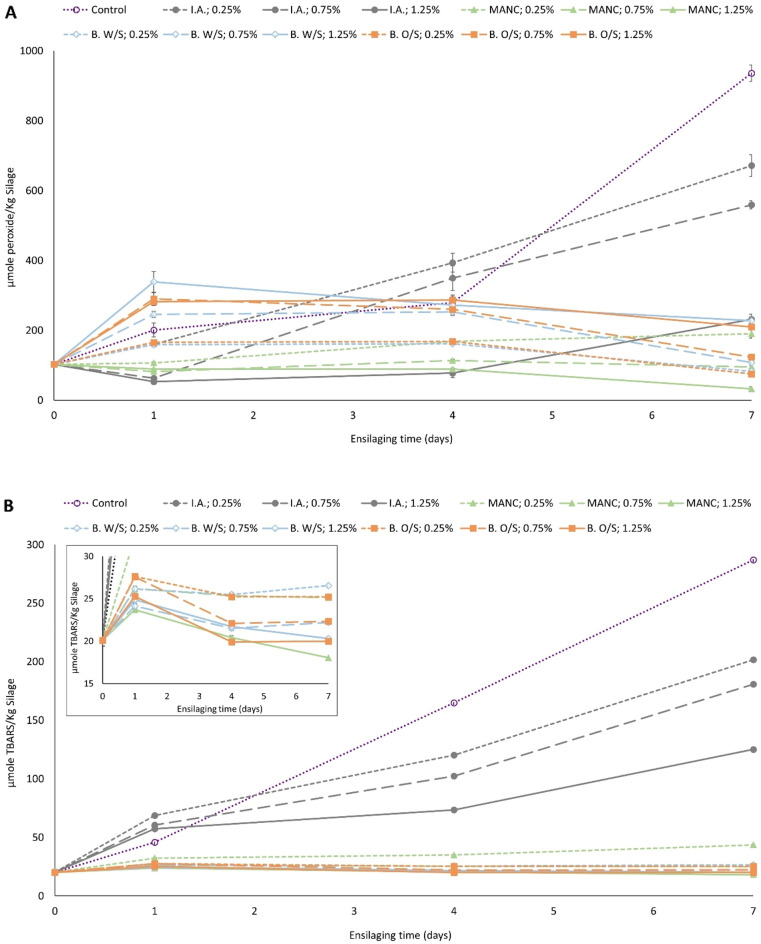
Effect of antioxidants in different concentrations on PV (**A**) and TBARS (**B**) during ensilaging of herring filleting co-products at 22 °C. Control refers to silage without any antioxidant addition; and, time point zero (i.e. day 0) refers to minced herring co-products before ensilaging. Herring filleting co-products from batch-1 were used in this experiment. The inset in (**B**) shows the differences in TBARS on 15–30 µmole TBARS/Kg silage scale. Results are expressed as mean ± SEM (n = 3). *I.A.* isoascorbic acid, *MANC* Duralox MANC-213, *B. W/S* BORDANTIX W/S, *B. O/S* BORDANTIX O/S.

**Figure 3:**
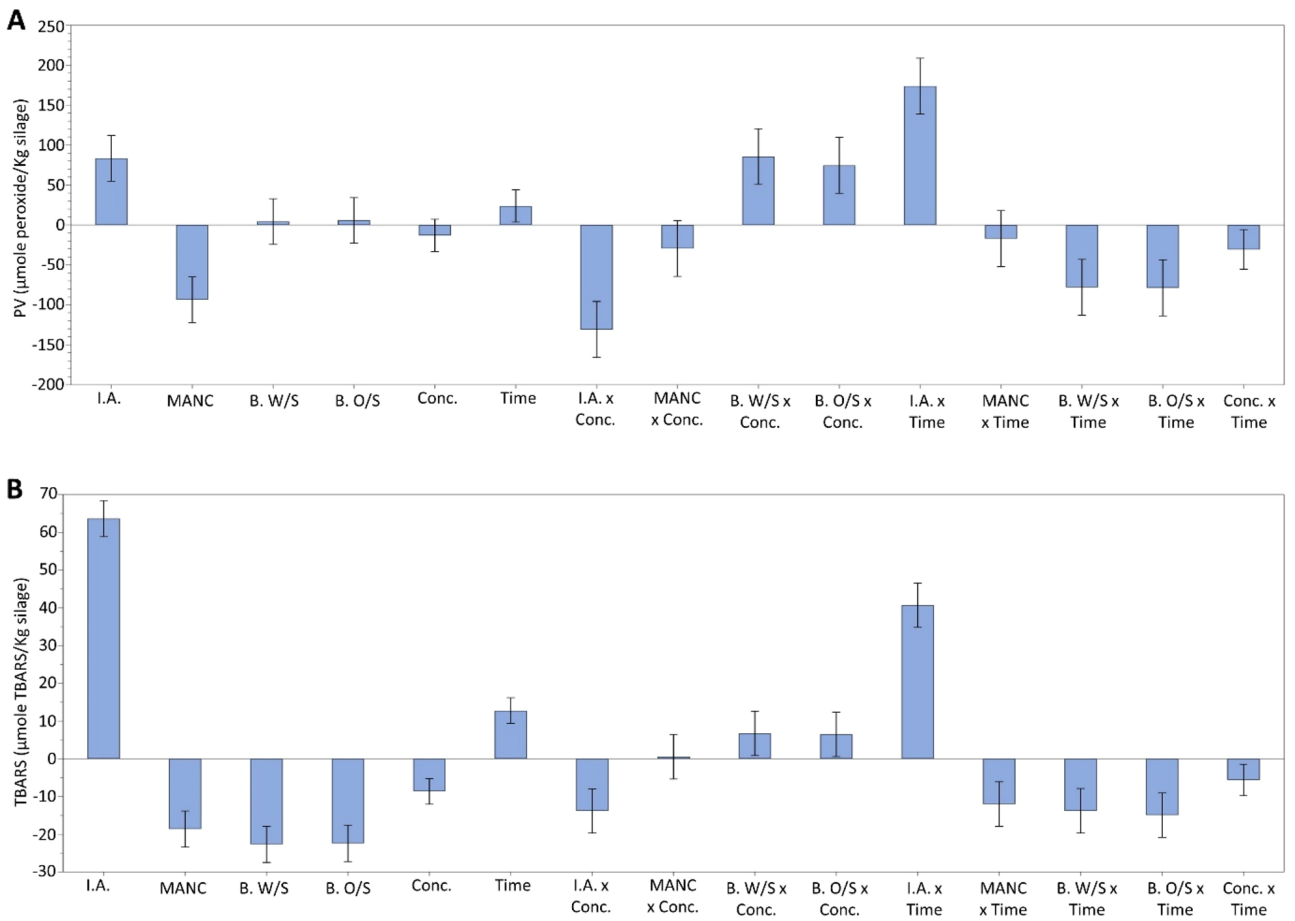
Coefficient plots showing responses of antioxidants in different concentrations on PV (**A**) and TBARS (**B**) during 0–7 days of ensilaging of herring filleting co-products at 22 °C. Coefficient plots shown in panel A and B refer to data presented in Fig. 2A,B, respectively. Responses were scaled and centered; and, the size of coefficients represents the change in their respective responses when a factor varies from medium to high level, while keeping other factors at their average values. *I.A.* isoascorbic acid, *MANC* Duralox MANC-213, *B. W/S* BORDANTIX W/S, *B. O/S* BORDANTIX O/S, *Conc.* concentration.

The effect of type and concentration of antioxidants on PV and TBARS inhibition during ensilaging of herring filleting co-products is shown in Figs. 2 and 3. Both PV and TBARS increased significantly (p < 0.05) over time in silage without antioxidant added (i.e. control), and this sample had the highest levels of both PV and TBARS at the end of ensilaging, i.e. 935.95 µmole peroxide/kg silage and 286.99 µmole TBARS/kg silage, respectively. Isoascorbic acid decreased PV and TBARS build-up in a concentration-dependent manner; i.e. PV and TBARS decreased in the order of 671.51 > 559.34 > 231.40 µmole peroxide/kg silage and 201.61 > 180.63 > 125.04 µmole TBARS/Kg silage, respectively, after 7 days with an increase in isoascorbic acid concentration from 0.25, 0.75 and 1.25% (Fig. 2A,B). Both BORDANTIX W/S and BORDANTIX O/S were equally effective in PV and TBARS inhibition at all three concentrations, and there were no significant (p > 0.05) differences between these two antioxidants. However, after 7 days the PV increased from 82.64 to 227.38 and 74.86 to 209.85 µmole peroxide/kg silage in samples containing BORDANTIX W/S and BORDANTIX O/S, respectively, with an increase in concentration from 0.25 to 1.25%, while TBARS at this time point decreased from 26.53 and 20.30 to 25.20 and 19.97 µmole TBARS/kg silage with these additions (Fig. 2A,B). At the end of 7 days ensilaging, BORDANTIX-fortified samples were however significantly (p < 0.05) less oxidized than the control, both in terms of PV and TBARS. Duralox MANC-213, in contrast, lowered both PV and TBARS values down to 190.05–32.38 µmole peroxide/kg silage and 43.53–18.04 µmole TBARS/kg silage, respectively, with an increase in concentration from 0.25 to 1.25% (Fig. 2A,B). Overall, Duralox MANC-213 thus provided the highest protection against both PV and TBARS development, while BORDANTIX antioxidants were effective primarily against TBARS development (Fig. 3).

In a separate trial, using a new batch of herring co-products, the two classic synthetic antioxidants BHT and propyl gallate were investigated for comparative purposes (see Supporting Information; Fig. S1). Both provided significantly (p < 0.05) lower TBARS levels during ensilaging, compared to control; and, there were no significant (p > 0.05) differences between them at all three inclusion levels. A higher concentration provided a better TBARS inhibition; for example, increasing concentration from 0.25 to 1.25% lowered TBARS values at day 7 from 76.86 to 50.50 as well as from 59.60 to 31.53 µmole TBARS/Kg silage, for BHT and propyl gallate, respectively. At the 1.25% inclusion level, BHT and propyl gallate had thus reduced the TBARS value, compared to the control, by 92.61 and 95.38% at day 7. Further, under the same conditions, isoascorbic acid, BORDANTIX W/S, BORDANTIX O/S and Duralox-MANC-213 reduced the TBARS-values by 56.43, 92.92, 93.04, and 93.71%, respectively.

The increasing PV and TBARS values in controls during 7 days of ensilaging suggest that the herring’s endogenous antioxidants such as α-tocopherol and ascorbic acid^35–37^, as well as low molecular weight (LMW) peptides produced during the ensilaging^5^, were not sufficient in inhibiting lipid oxidation. Possible reasons for this could be due to the fact that both ascorbic acid and tocopherol are quickly consumed during storage and processing of herring, even at cold temperatures^12,13,38^, while the peptides primarily act by chelating metal ions^39^, which are not main pro-oxidants in the herring silage^15^. α-Tocopherol provides antioxidative protection by scavenging lipid peroxyl and alkoxyl radicals, with the formed tocopherol radical then being reduced to α-tocopherol by ascorbic acid^40^. The latter can also scavenge radicals or reduce hypervalent or heme iron^41^. Isoascorbic acid provided some protection against oxidation, particularly at the higher concentration, which was most likely due to scavenging of free radicals and/or reducing hypervalent iron^22^ or heme iron^41^. However, since there was substantial TBARS development, even after 1.25% addition, isoascorbic acid alone would not be recommended as the only antioxidative protection during ensilaging.

All three rosemary extract-based antioxidants provided equally good protection at 0.25%, 0.75% and 1.25% inclusions; however, Duralox MANC-213, was most efficient in both PV and TBARS inhibition. Duralox MANC-213 is a mixture of rosemary extract, tocopherols, ascorbic acid, and citric acid; with the former containing several different phenolic compounds like rosmarinic acid, carnosol, and carnosic acid. The latter two are efficient radical scavenging o-diphenols^40^ and the most active components of rosemary extract, ascribed more than 90% of the antioxidative properties of the extract^42^.Carnosol and carnosic acid provide antioxidative protection by scavenging free radicals; for example, it has been reported that the 12- or 14-position of carnosic acid reacts with free radicals e.g. lipid peroxyl radicals to form stable products by an oxidative coupling reaction^24^. Also, the carnosol of rosemary extract can work as lipoxygenase (LOX) inhibitor^43^, the latter being a well-known pro-oxidant in muscle foods^44^. Further, carnosol and carnosic acid have been reported to provide better antioxidative protection at pH 4.0 than at pH 7.0, in a corn oil-in-water emulsion oxidized at 60 °C for 4 days^25^. Citric acid works as metal chelator^40^, while ascorbic acid, as isoascorbic acid, works by e.g. scavenging free radicals, quenching active oxygen forms, and regeneration of primary antioxidants; for example it has been reported to provide excellent synergism with e.g. citric acid, tocopherol, and metal chelators^45^. Besides multiple mechanisms of action, the fact that Duralox MANC-213 contains both hydrophilic and lipophilic antioxidants is expected to aid its partitioning both into the oil phase and oil–water interface under acidic ensilaging conditions^25^. In addition, our recent study revealed that Duralox MANC-213 specifically prevented Hb autoxidation and heme-loss^21^, which is expected to be an important antioxidative mechanism in the herring silage.

BORDANTIX LIQUID W/S is a natural extract of rosemary containing 5.3% carnosic acid, in addition to carnosol, rosmarinic acid, rosmaridiphenol, rosmaridiquinone, and rosmanol, and, is soluble in both water and oil. BORDANTIX LIQUID O/S contains the same components as the W/S variety, while its carnosic acid content is 5.2% and it is soluble mainly in oil. The antioxidative protection of both BORDANTIX antioxidants is most probably derived from their carnosic acid and carnosol content^42^. The increased PV values observed with increased BORDANTIX concentrations could possibly be due to its rosmarinic acid which can work as a pro-oxidant by generating free radicals and H_2_O_2_^46^, or, by preventing lipid hydroperoxide breakdown, for example by stabilizing heme/Hb.

The two synthetic antioxidants BHT and propyl gallate provide antioxidative protection by donating hydrogen to free-radicals while at the same time being converted to very stable radical intermediates^40^. Although both BHT and propyl gallate are classified as “generally recognized as safe (GRAS)” and allowed in foods and food packaging in low concentrations, concerns have been raised in the past decades due to their adverse health effects^23^, which is why food and feed producers today normally seek other options, preferably natural antioxidants. Further, several recent studies reported protection of fish feed, produced from marine raw material, against lipid oxidation using natural antioxidants e.g. rosemary extract^47,48^. Along this line, the ensilaging trials in this study revealed that higher antioxidant concentrations gave lower TBARS values; and, apart from isoascorbic acid, the gain in quality was largest from 0.0 to 0.25% antioxidant, while increasing the concentration further from 0.75% to 1.25% only lowered TBARS sparsely. Thus, around 0.75% rosemary-containing antioxidant addition appears sufficient to provide protection against lipid oxidation during ensilaging of herring filleting co-products at 22 °C, while for isoascorbic acid, levels > 1.25% would be needed. The next step would be to evaluate how the antioxidants affect the sensory properties of the silages, both in a feed and food scenarios.

From a health perspective, it is important to stress that Atlantic salmon does not seem to discriminate oxidized feeds, but intake of oxidized diets has shown to increase TBARS in plasma of salmon up to fourfold^49^, potentially leading to a reduced fish health. The synthetic antioxidant etoxyquin was earlier widely used by the fish feed industry until it was suspended in 2017 by the EU commission, and has mainly been replaced by e.g. butylated hydroxyanisole (BHA) and BHT^47,48^. However, as mentioned above, natural antioxidants are attractive to both feed and food producers for clean-label reasons.

### The effect of antioxidants on PV and TBARS during heat-treatment of silages

Silage needs to be heat-treated to prevent an excessive formation of FAA and TVB-N, inactivate thiaminases, and mitigate pathogen risks. However, it is well-known that heat-treatment can affect the status and activity of many pro-oxidants; for example, heme or low molecular weight transition metal ions (iron) might be released from proteins during heat-treatment and catalyze lipid oxidation^50^. Thus, heat-stable antioxidants should be used to provide maximum protection of the silage. Both BHT and propyl gallate have long been used in processes involving heat-treatments^51^. Further, rosemary extract has also been reported to provide antioxidative protection during thermal treatment (90 °C for 50 min) of gels prepared form Atlantic mackerel (*Scomber scombrus*)^26^. We therefore assumed that these antioxidants would provide protection during heat-treatment of silage. Isoascorbic acid, on the other hand, was left out for this step as it did not provide sufficient protection against lipid oxidation during ensilaging.

**Figure 4:**
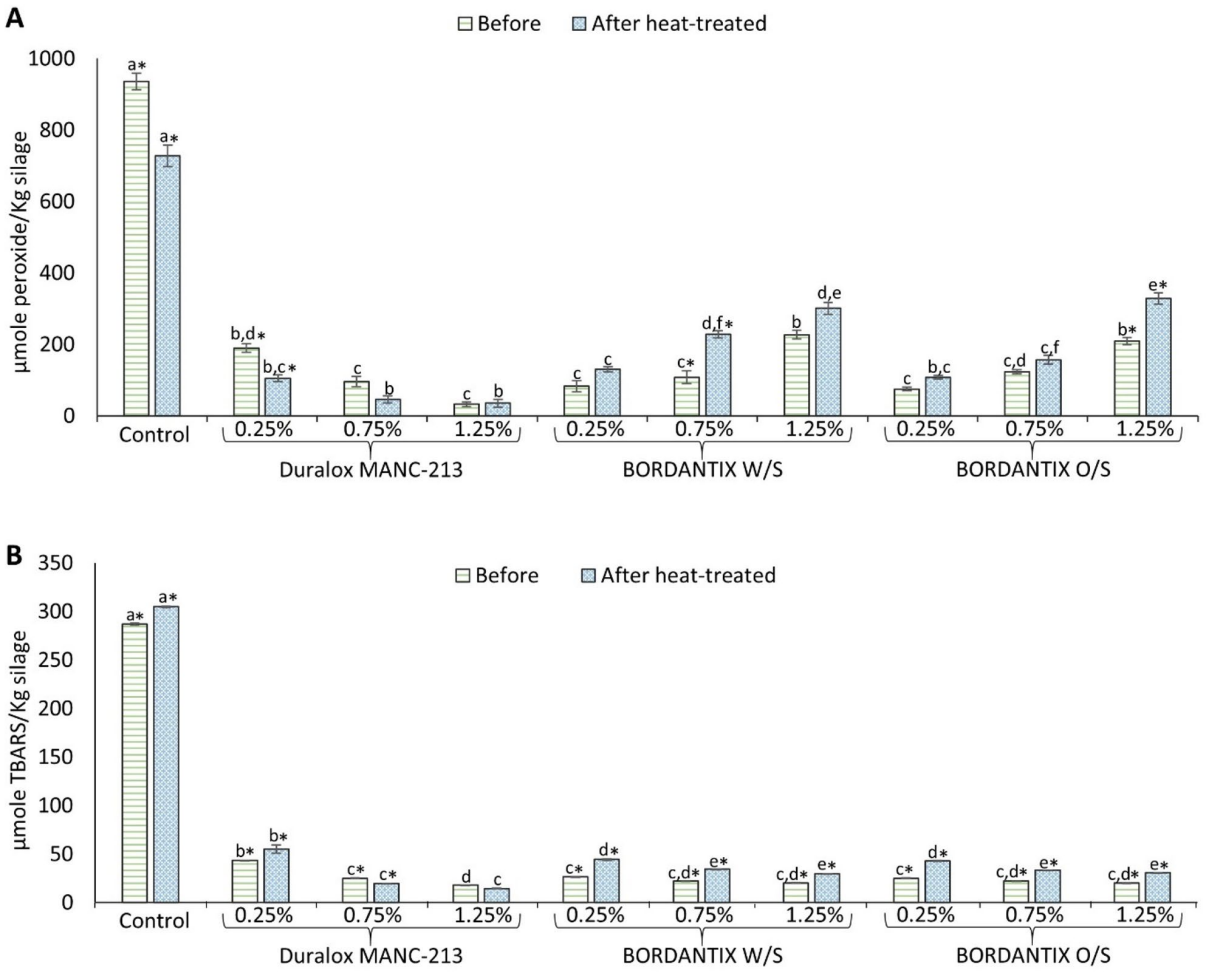
Effect of antioxidants in different concentrations on PV (**A**) and TBARS (**B**) before and after heat-treating the silage at 85 °C for 30 min. Control refers to silage without any antioxidant addition. Herring filleting co-products from batch-1 were used in this experiment. Star (*) sign represents significant (p < 0.05) difference between silage samples before and after heat-treatment; and, different lower-case letters either before and after heat-treatment denote significance (p < 0.05) difference. Results are expressed as mean ± SEM (n = 3).

The effect of heat-treating the final silage product at 85 °C for 30 min on PV and TBARS is shown in Fig. 4. The PV of control silage decreased significantly (p < 0.05) from 935.95 to 728.03 µmole peroxide/Kg silage after heat-treatment (Fig. 4A), while the TBARS value increased significantly (p < 0.05) from 286.99 to 304.69 µmole TBARS/Kg silage (Fig. 4B). Similar trends were also noticed for silage containing 0.25% Duralox MANC-213 (Fig. 4A,B). However, no significant (p > 0.05) changes in PV after heat-treatment of silages containing 0.75% and 1.25% Duralox MANC-213 were noticed. Also, there was no significant (p > 0.05) change in TBARS in silage containing 1.25% Duralox MANC-213; however, TBARS value decreased significantly (p < 0.05) in silage containing 0.75% Duralox MANC-213 after heat-treatment. In case of BORDANTIX antioxidants, increased PV and TBARS values were noticed after heat-treatment at all three concentrations (Fig. 4A,B). PV of the control silage from batch-2 filleting co-products behaved very different from that in batch-1 co-products, and significantly (p < 0.05) increased after heat-treatment (see Supporting Information; Fig. S2). TBARS on the other hand significantly (p < 0.05) decreased (see Supporting Information; Fig. S2). Apart from these, there were no significant (p > 0.05) differences in PV and TBARS after heat-treatment of silages containing either BHT or propyl gallate at three different concentrations (see Supporting Information; Fig. S2).

The lower PV values after heat-treatment are probably resulting from the breakdown of temperature-sensitive peroxides in the presence of heme proteins^12^, which in turn leads to increased TBARS values. However, an increasing trend for PV was also noticed in this study, which can be explained by the fact that some peroxides are being formed during heat-treatment and/or that peroxide breaking species, such as heme, were stabilized by phenolic compounds^52,53^. Lower TBARS values after heat-treatment could be due to breakdown of malondialdehyde (MDA)—the main carbonyl compound responding in the TBARS test—into acetaldehyde and formic acid, and/or further reaction between MDA and amino acids/peptides/proteins to form non-enzymatic browning products, which were not detected in the TBARS test^12^. Non-enzymatic browning reaction products can however provide some antioxidative protection^54^. Overall, silage with lower PV and TBARS before heat-treatment—i.e. silage containing higher antioxidant concentrations—had relatively lower PV and TBARS after heat-treatment; suggesting that adequate concentration of antioxidant should be added at the start of ensilaging to provide better protection in later process steps.

### Effect of antioxidants on TBARS during storage of silage at two different temperatures

**Figure 5:**
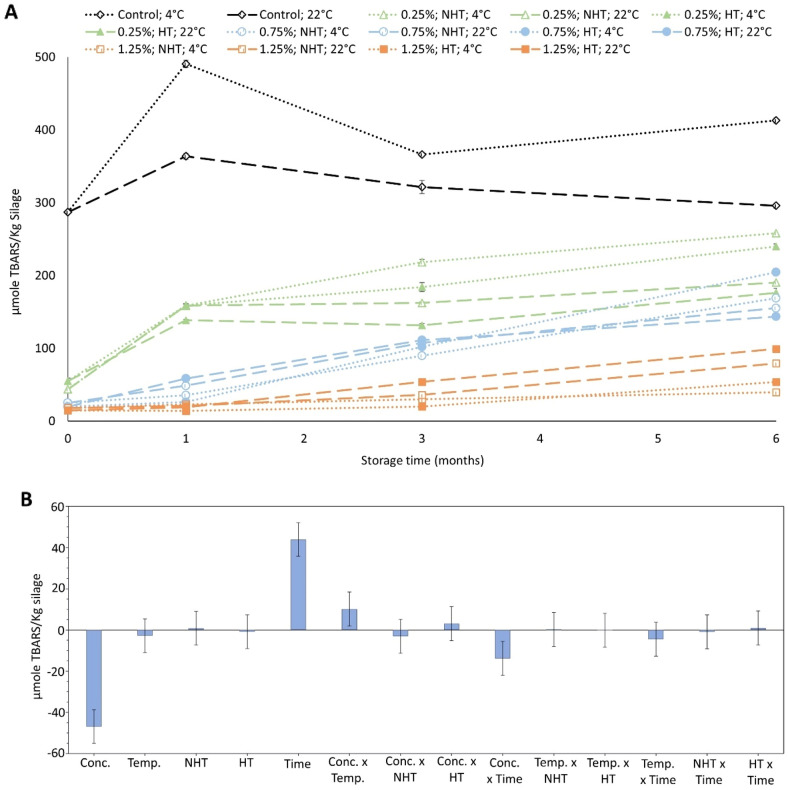
Effect of storage temperature, time, heat-treatment, and Duralox MANC-213 concentrations on TBARS (**A,B**). Control refers to silage without any antioxidant addition. Herring filleting co-products from batch-1 were used in this experiment. Results are expressed as mean ± SEM (n = 3). Coefficient plot (**B**), refers to data presented in (**A**), shows responses of antioxidant concentrations, heat-treatment, storage temperature and time on TBARS. Responses were scaled and centered; and, the size of coefficients represents the change in their respective responses when a factor varies from medium to high level, while keeping other factors at their average values. *NHT* non-heat-treated, *HT* heat-treated, *Conc.* concentration, *Temp*. temperature.

Since Duralox MANC-213 was considered the most promising antioxidant during ensilaging and heating, silages with this antioxidant at 0.25–1.25% levels were taken further to a 6-month storage trial at 4 °C and 22 °C. As can be seen in Fig. 5A,B, TBARS values increased significantly (p < 0.05) with storage time, and significantly (p < 0.05) lower TBARS values were noticed in silages containing higher antioxidant concentrations. Also, heat-treated silages containing 0.25% and 0.75% Duralox MANC-213, stored at 22 °C, had lower TBARS values compared to the ones stored at 4 °C. Contrary, heat-treated silage containing 1.25% Duralox MANC-213 stored at 22 °C had higher TBARS values, compared to the one stored at 4 °C. Similar trends were also noticed for non-heat-treated silage stored at both 4 °C and 22 °C. Overall, TBARS values increased significantly (p < 0.05) with storage time, while a higher antioxidant concentration gave significantly (p < 0.05) lower TBARS values (Fig. 5B).

Based on the PLS analysis, no significant (p > 0.05) effect of heat-treatment and storage temperature on TBARS was noticed in this study; rather, the storage time per se played the most important role in TBARS development (Fig. 5B). Lipoxygenase is a well-known pro-oxidant in muscle foods^44^, and heat-treatment at 80 °C for 5 min have been reported to completely inactivate its activity in lake herring (*Coregonus artedi*)^55^. Besides, the carnosol of rosemary extract has been reported to work as lipoxygenase inhibitor^43^. Thus, lower TBARS values were expected in Duralox MANC-213-fortified heat-treated silages, compared to non-heat-treated ones. However, this was not the case here, suggesting that lipoxygenase was not a major prooxidant in herring silages. In general, higher antioxidant concentrations provided better oxidative stability over storage (Fig. 5B), suggesting that both added and endogenous antioxidants are consumed over time^12^.

Overall, the TBARS values of silage containing e.g. 0.75% Duralox MANC-213 after 7 days of ensilaging and heat-treatment were 25.12 and 19.76 µmole TBARS/kg silage, respectively. The TBARS values during 0–6 months storage both at 4 °C or 22 °C were within the range of 19.76–204.63 µmole TBARS/Kg silage. These values were similar, or in some cases well below, to the reported TBARS values of e.g. Atlantic mackerel (*Scomber scombrus*) fillets containing antioxidants (either sodium erythorbate or phosphate) and stored at − 25 °C for 0–15 months^56^, suggesting that silage has comparable oxidative quality to that of Atlantic mackerel fillets. Besides, frozen and thawed herring filleting co-products were used in this study, which usually gives higher TBARS values than fresh co-products^5^, probably due to an increased release of both pro-oxidative low molecular weight iron and copper^44^. However, in case of industrial-scale application, ensilaging can be done with fresh co-products generated right after filleting, which will probably give even lower TBARS values, further improving the quality of silage.

### Effect of heat-treatment, storage temperature and time on DH, FAA, and TVB-N

**Figure 6:**
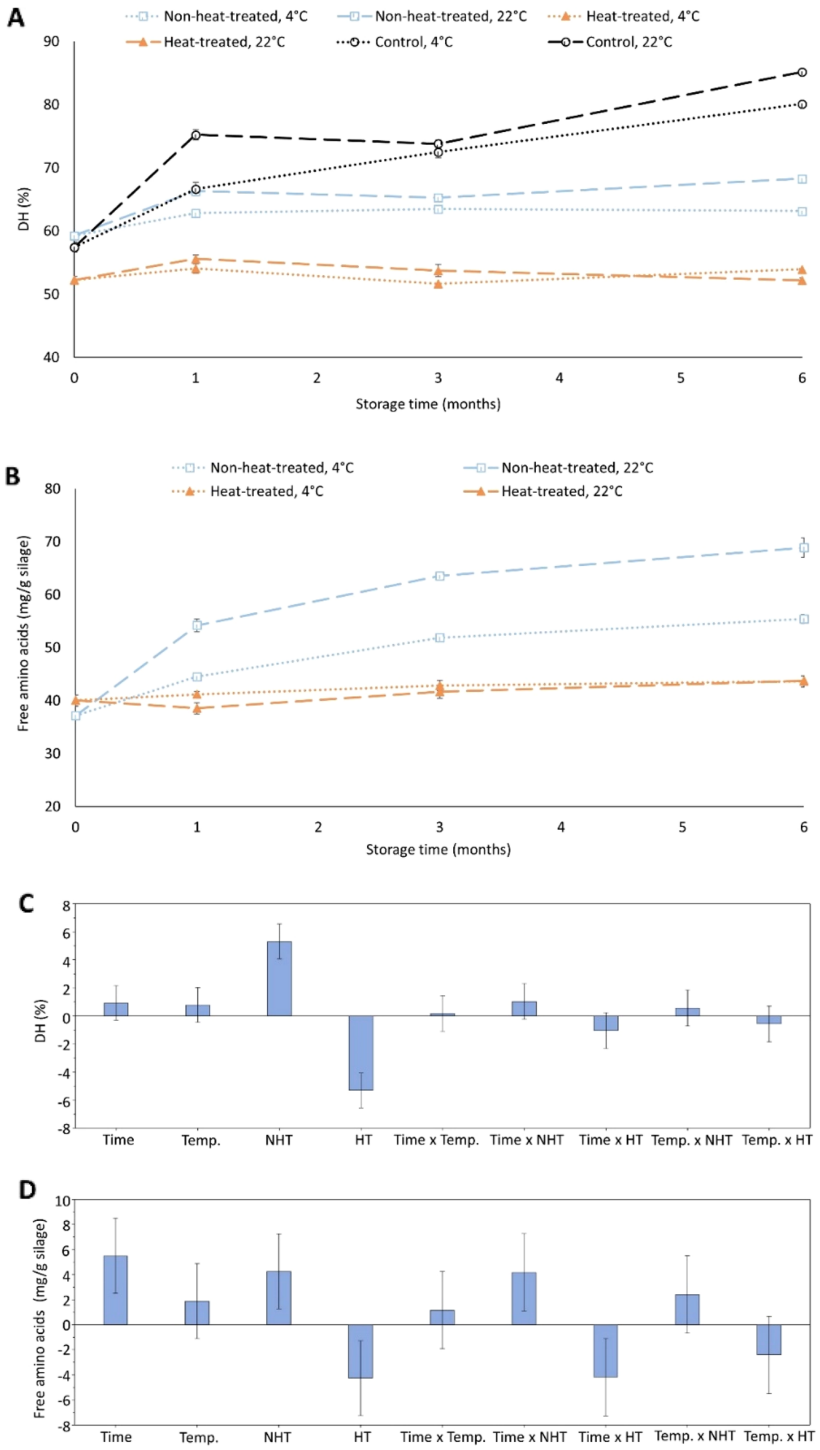
Effect of heat-treatment, storage temperature and time on DH (**A,C**) and free amino acids (**B,D**) in silage samples containing 0.75% w/w Duralox MANC-213. Control refers to silage without any antioxidant addition. Herring filleting co-products from batch-1 were used in this experiment. Results are expressed as mean ± SEM (n = 2). Coefficient plots shown in (**C,D**) refer to data presented in (**A,B**), respectively. Responses were scaled and centered; and, the size of coefficients represents the change in their respective responses when a factor varies from medium to high level, while keeping other factors at their average values. *NHT* non-heat-treated, *HT* heat-treated, *Temp*. temperature.

There were no significant (p > 0.05) changes in DH and FAA of heat-treated silage during storage (Fig. 6A–D), and, the storage temperature had no significant (p > 0.05) effect either. Contrary, both DH and FAA of non-heat-treated silage significantly (p < 0.05) increased over time, and storage at 22 °C generated significantly (p < 0.05) higher DH and FAA, compared to the storage at 4 °C (Fig. 6A,B). PLS analysis revealed that the heat-treatment, at large, controlled DH and FAA more than storage temperature and time (Fig. 6C,D). The fact that heat-treatment would inactivate proteases was expected^57^, thereby stopping autolysis during subsequent storage. The observed lower DH values at 0 month in heat-treated silages, compared to non-heat-treated ones, could possibly be due to changes of primary amino groups upon heat-treatment, e.g. by forming Maillard reaction products such as pyrroles^33,54^, preventing their reaction with the o-phthaldialdehyde (OPA)-reagent^33^. The slower autolysis rate at 4 °C in non-heat-treated silage, compared to at 22 °C, was in line with our earlier findings and suggests that endogenous proteases from herring were less active at lower temperatures^5^. The DH increase levelled off somewhat after ~ 1 month at both 4 °C and 22 °C (Fig. 6A) could be due to lack of favorable binding/splitting sites on the substrates^8^.

**Figure 7:**
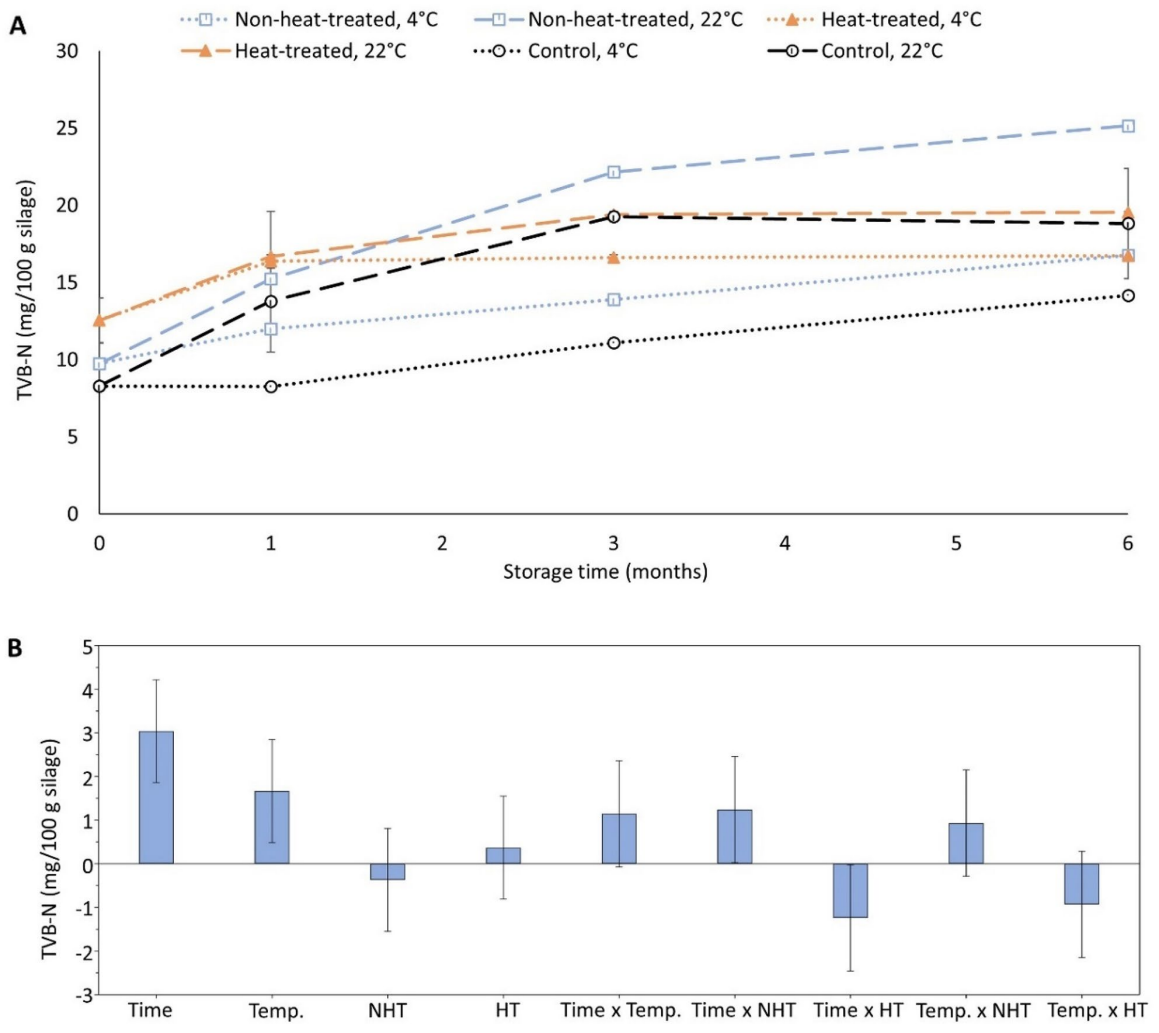
Effect of heat-treatment, storage temperature and time on TVB-N in silage samples containing 0.75% w/w Duralox MANC-213 (**A,B**). Control refers to silage without any antioxidant addition. Herring filleting co-products from batch-1 were used in this experiment. Results are expressed as mean ± SEM (n = 2). Coefficient plot (**B**), refers to data presented in (**A**), shows responses of heat-treatment, storage temperature and time on TVB-N. Responses were scaled and centered; and, the size of coefficients represents the change in their respective responses when a factor varies from medium to high level, while keeping other factors at their average values. *NHT* non-heat-treated, *HT* heat-treated, *Temp*. temperature.

TVB-N, which measures degradation of proteins and non-protein nitrogenous compounds into volatile amines such as trimethylamine nitrogen (TMA-N) and NH_3_^28^, has been widely used as a quality indicator of fish and fish products. TVB-N values in this study significantly (p < 0.05) increased over storage time and temperature (Fig. 7A,B), which was in line with earlier studies^5,28,58^. Further, higher TVB-N values were noticed in non-heat-treated silage than heat-treated ones stored at 22 °C, suggesting that continuous autolysis (Fig. 6A) contributed e.g. to formation of NH_3_ by deamination of amide-N groups containing amino acids such as asparagine and glutamine into NH_3_^58^. TVB-N values recorded in this study were well below the acceptable limit of 30 mg TVB-N per 100 g fish for human consumption^28^, which was different compared to previously reported high TVB-N values in silage prepared from different combinations of plaice (*Pleuronectes platessa*), sole (*Solea solea*), flounder (*Platichthys flesus*), and whiting (*Merlangius merlangus*) species^58^. This could probably be due to a good starting quality of the herring co-products used for ensilaging. Besides, the rosemary extract, one of the components of Duralox MANC-213, has been reported to provide antimicrobial effect^59^; however, in this study, no significant (p > 0.05) differences in TVB-N values were noticed in Duralox MANC-213-fortified silages, compared to the controls. Overall, the fact that TVB-N values were lower than the limit acceptable for human consumption, even after 6 months storage at both 4 °C and 22 °C, provides high flexibility regarding transportation of silage from ensilaging plant to further value-added processing plant.

Although the addition of rosemary extract-based antioxidants efficiently minimized lipid oxidation during ensilaging, heat treatment and subsequent storage of silage, it should be mentioned that the silages obtained a weak rosemary flavor. Further studies are therefore required to investigate the consumer acceptance of such products and/or masking of the rosemary flavor when added to different food matrices such as drinks, soups, sauces, sports nutrition products etc. In this context, legislations around maximum allowed dosages of the components found in the tested rosemary derived antioxidant mixtures indeed must also to be considered before scaling up to industrial scale.

## Conclusion

The effect of rosemary extract-based antioxidants on lipid oxidation during ensilaging, heat-treatment and subsequent storage has been reported here for the first time. Among the antioxidants studied, Duralox MANC-213 provided the best protection against both PV and TBARS development during ensilaging and heat-treatment, with the two highest antioxidant concentrations (0.75 and 1.25%) reducing lipid oxidation the most. The fact that silages with low TBARS values before heat-treatment had lower TBARS values after heat-treatment, suggests that adequate levels of antioxidants should be added at the beginning of ensilaging to keep the oxidation level to a minimum. During prolonged storage at 4 °C, the highest antioxidant concentration (1.25%) could prevent TBARS development during 6 months almost completely, while a level > 1.25% might be needed to prevent TBARS formation at 22 °C. Heat-treating the silage stopped autolysis and thus prevented an excessive formation of FAA during extended storage of the silage. TVB-N values of all silages during the 6-month storage were well below 30 mg TVB-N per 100 g fish sample, which is an acceptable limit for human consumption. Overall, the results of this study suggest that herring silage produced in the presence of natural antioxidants and with enzymes heat-inactivated after reaching a desired DH% level has a very low level of secondary lipid oxidation products which can pave the way for using silage not only in high-value feed applications, but also in food.

## Supplementary Information

Supplementary Information.
