# Supplementary material for: Effect of antioxidants on lipid oxidation in herring (Clupea harengus) co-product silage during its production, heat-treatment and storage
Source: Sci Rep. 2022 Mar 1;12:3362. doi: 10.1038/s41598-022-07409-8 (PMC8888572; doi:10.1038/s41598-022-07409-8)
Supplement: Supplementary file 1 — Supplementary Information. [file 41598_2022_7409_MOESM1_ESM.docx]

**Supporting information**

**Effect of antioxidants on lipid oxidation in herring (*Clupea harengus*) co-product silage during its production, heat-treatment and storage**

Mursalin Sajib^a^*, Markus Langeland^b^ and Ingrid Undeland^a^

^a^Food and Nutrition Science, Department of Biology and Biological Engineering, Chalmers University of Technology, SE-41296 Gothenburg, Sweden

^b^Department of Animal Nutrition and Management, Swedish University of Agricultural Sciences, SE-75007 Uppsala, Sweden

*Corresponding Author: E-mail: mursalin@chalmers.se; Tel: +4631 772 68 63


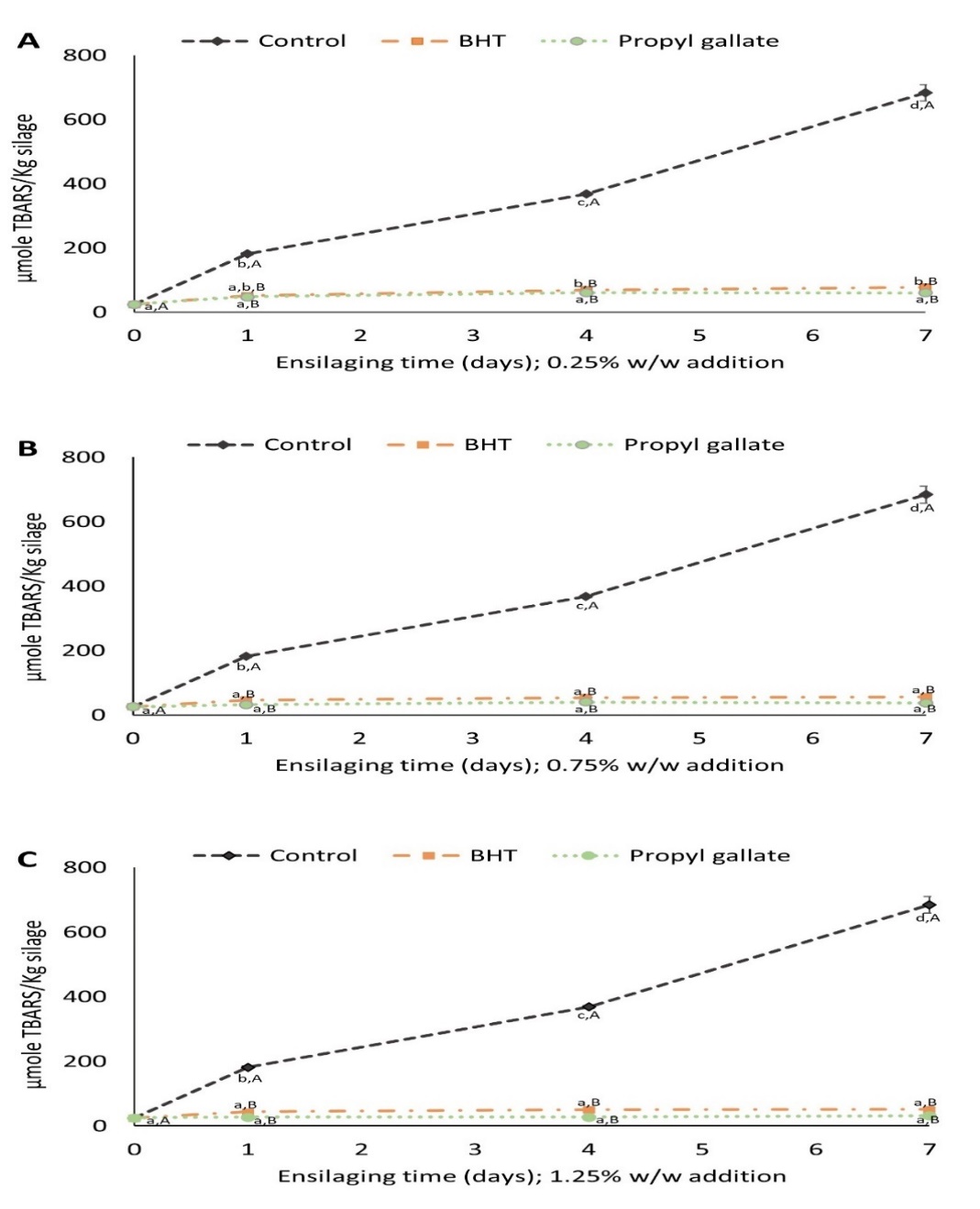


**Supplementary figure S1.** Effect of BHT and propyl gallate on TBARS; (A) 0.25% w/w, (B) 0.75% w/w, and, (C) 1.25% w/w addition of antioxidants. Control refers to silage without any antioxidant addition; and, time point zero (i.e. day 0) refers to minced herring co-products before ensilaging. Herring filleting co-products from batch-2 was used in this experiment. Data points with different lower-case letter for individual antioxidants/control are significantly (p < 0.05) different from each other; and data points with different upper-case letter for the same time point among different antioxidants/control are significantly (p < 0.05) different from each other. Results are expressed as mean ± SEM (n = 3).


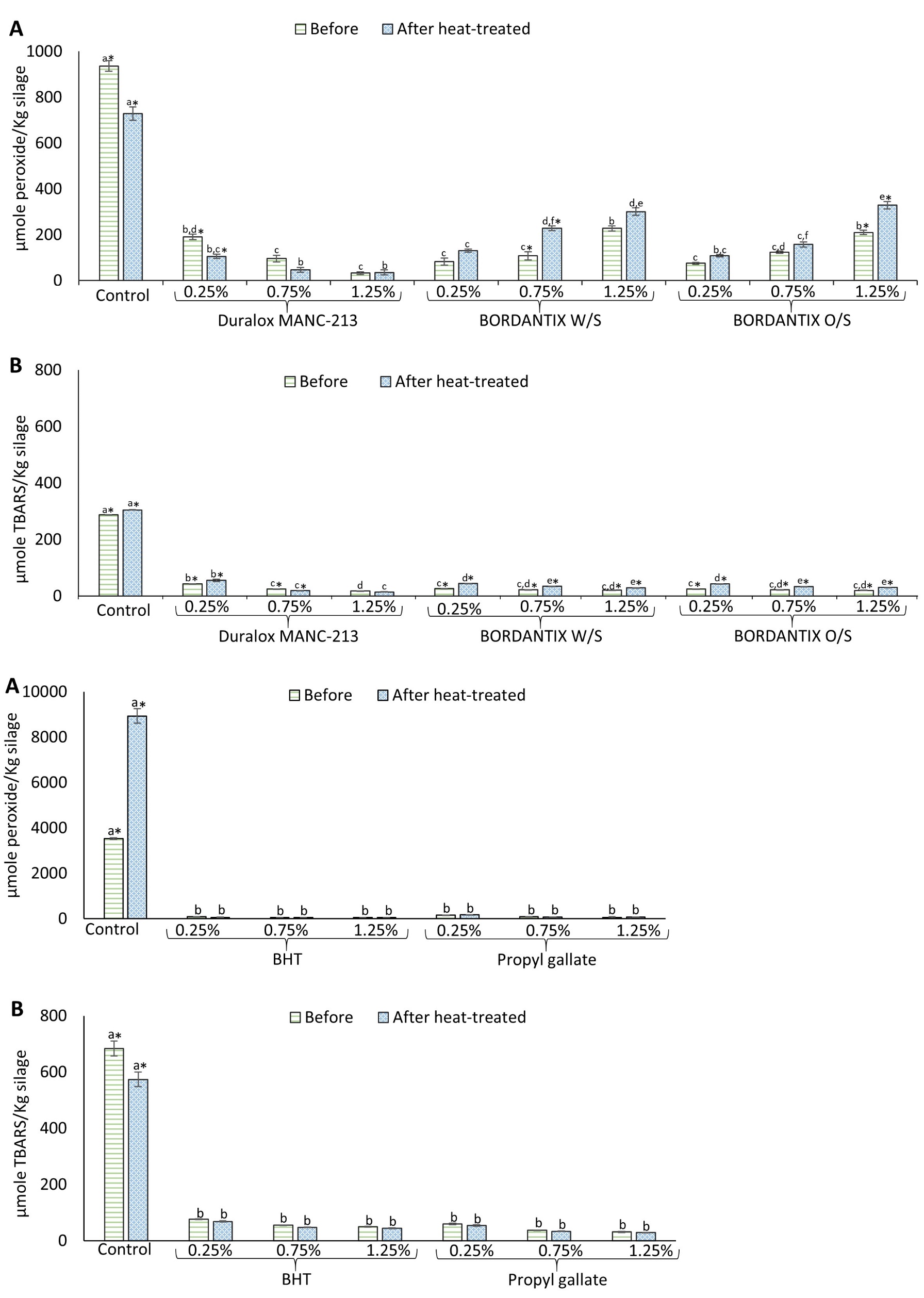


**Supplementary figure S2.** Effect of antioxidants in different concentrations on PV (A) and TBARS (B) before and after heat-treating the silage at 85°C for 30 min. Control refers to silage without any antioxidant addition. Herring filleting co-products from batch-2 was used in this experiment. Star (*) sign represents significant (p < 0.05) difference between silage samples before and after heat-treatment; and, different lower-case letters before and after heat-treatment denote significance (p < 0.05) difference. Results are expressed as mean ± SEM (n = 3).

**Table S1. Changes in PV and TBARS after heat-treating the silage at 85°C for 30 min**

| Co-product batch | Antioxidant | Concentration | PV (%) | TBARS (%) |
| --- | --- | --- | --- | --- |
| Batch 1 | Control | None | -28.56 | 5.81 |
|  | Duralox MANC-213 | 0.25% | -80.99 | 20.88 |
|  |  | 0.75% | -107.47 | -27.08 |
|  |  | 1.25% | 8.11 | -23.91 |
|  | BORDANTIX W/S | 0.25% | 36.57 | 40.28 |
|  |  | 0.75% | 52.75 | 35.50 |
|  |  | 1.25% | 24.39 | 31.36 |
|  | BORDANTIX O/S | 0.25% | 30.83 | 41.27 |
|  |  | 0.75% | 21.47 | 33.15 |
|  |  | 1.25% | 36.14 | 34.66 |
| Batch 2 | Control | None | 60.47 | -19.13 |
|  | BHT | 0.25% | -32.11 | -12.27 |
|  |  | 0.75% | -9.89 | -16.34 |
|  |  | 1.25% | -8.62 | -14.60 |
|  | Propyl gallate | 0.25% | 8.39 | -10.14 |
|  |  | 0.75% | -20.46 | -8.58 |
|  |  | 1.25% | 21.85 | -9.23 |

A positive and negative value indicates increase and decrease in PV/TBARS after heat-treatment, respectively. Percentage (%) changes in PV and TBARS after heat-treatment were calculated from mean values (n = 3) using the formula below:

Change = ((Value after heat-treatment – Value before heat-treatment)/ Value after heat-treatment) * 100
